# Fragile, unfaithful and persistent Ys—on how meiosis can shape sex chromosome evolution

**DOI:** 10.1038/s41437-022-00532-2

**Published:** 2022-04-22

**Authors:** Aurora Ruiz-Herrera, Paul D. Waters

**Affiliations:** 1 Genome Integrity and Instability Group, Institut de Biotecnologia i Biomedicina (IBB), Universitat Autònoma de Barcelona (UAB), Cerdanyola del Vallès, 08193 Spain; 2 Departament de Biologia Cel·lular, Fisiologia i Immunologia, Universitat Autònoma de Barcelona (UAB), Cerdanyola del Vallès, 08193 Spain; 3 School of Biotechnology and Biomolecular Sciences, Faculty of Science, UNSW Sydney, Sydney, NSW 2052 Australia

**Keywords:** Sexual selection, Genome, Cytogenetics, Evolutionary biology

## Abstract

Sex-linked inheritance is a stark exception to Mendel’s Laws of Heredity. Here we discuss how the evolution of heteromorphic sex chromosomes (mainly the Y) has been shaped by the intricacies of the meiotic programme. We propose that persistence of Y chromosomes in distantly related mammalian phylogroups can be explained in the context of pseudoautosomal region (PAR) size, meiotic pairing strategies, and the presence of Y-borne executioner genes that regulate meiotic sex chromosome inactivation. We hypothesise that variation in PAR size can be an important driver for the evolution of recombination frequencies genome wide, imposing constraints on Y fate. If small PAR size compromises XY segregation during male meiosis, the stress of producing aneuploid gametes could drive function away from the Y (i.e., a fragile Y). The Y chromosome can avoid fragility either by acquiring an achiasmatic meiotic XY pairing strategy to reduce aneuploid gamete production, or gain meiotic executioner protection (a persistent Y). Persistent Ys will then be under strong pressure to maintain high recombination rates in the PAR (and subsequently genome wide), as improper segregation has fatal consequences for germ cells. In the event that executioner protection is lost, the Y chromosome can be maintained in the population by either PAR rejuvenation (extension by addition of autosome material) or gaining achiasmatic meiotic pairing, the alternative is Y loss. Under this dynamic cyclic evolutionary scenario, understanding the meiotic programme in vertebrate and invertebrate species will be crucial to further understand the plasticity of the rise and fall of heteromorphic sex chromosomes.

## Sex chromosomes and recombination are exceptions to Mendelian inheritance

Mendel’s paramount observations can be considered one of the great discoveries in biology and are summarised into three laws of heredity: (i) Law of Independent Assortment; (ii) Law of Dominance; and (iii) Law of Segregation (Castle 1903). Under the first law (independent assortment), genes for different traits are inherited independently of one another. Under the second law (dominance), in crosses between homozygous parents for a contrasting character, only one character of the parent appears in the first generation. Finally, the third law (segregation) postulates that each egg or sperm cell receives just one of two copies of each chromosome during germ cell production, with the copy randomly allocated to gametes.

George Mendel himself soon realised that his observations in peas presented some limitations, and over the century and a half that followed his investigations exceptions to Mendelian inheritance have arisen. These include phenomena such as co-dominance, incomplete dominance, epistasis, pleiotropy and lethal alleles, among others (Castle 1903; Bateson 1909; Castle and Little 1910; Wright 1968; Stearns 2010). Sex-linked inheritance represents one of these exceptions, as genes carried on differentiated heteromorphic sex chromosomes show different inheritance patterns to those on autosomes (non-sex chromosomes). In addition, the sex-limited chromosome (the Y or W) does not undergo recombination, with the exception of the pseudoautosomal region (PAR) (Hinch et al. 2014; Raudsepp and Chowdhary 2015), so genes on them do not assort independently. This contrasts with autosomes, in which a recombination event occurs during the formation of germ cells (in a single generation) that can break gene assemblies that are physically linked.

Here we discuss how the intricacies of the meiotic programme have shaped the evolution of differentiated heteromorphic sex chromosomes (mainly the Y), resulting in exceptions to Mendelian inheritance. We walk through basic concepts of meiotic progression and chromosome dynamics in germ cells (meiocytes), coupled with a description of variation in recombination rates between phylogroups and the sexes (heterochiasmy). We then present an integrative hypothesis on how the cellular control of the meiotic programme and the mechanistic constraints of recombination can shape sex chromosome evolution. Although we focus mainly on mammals (i.e., system for which most information is available), our proposed model could apply to any differentiated XY or ZW sex chromosome system.

## Meiosis and recombination

**Fig. 1:**
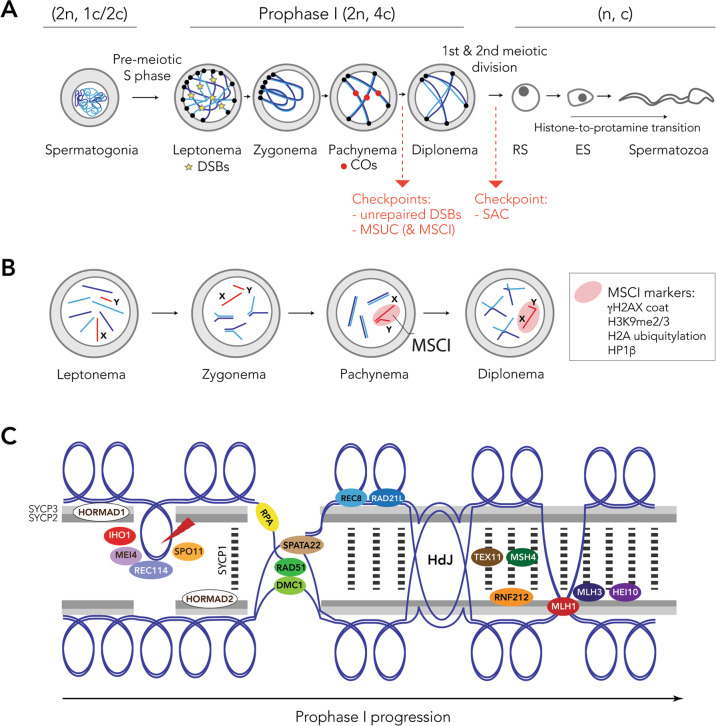
Schematic representation of spermatogenesis. **A** Stages of spermatogenesis. Spermatogonia commit to meiosis and after DNA duplication, primary spermatocytes undergo meiotic prophase I: leptonema, zygonema, pachynema and diplonema. Double-strand breaks (DBSs) are formed at leptonema, repaired in zygonema, resulting in crossovers (COs) in pachynema. Two meiotic checkpoints are activated in this stage: (i) response to unrepaired DSBs; and (ii) MSUC (meiotic silencing of unsynapsed chromatin) and MSCI (meiotic sex chromosome inactivation). The first and second meiotic divisions result in secondary spermatocytes and round spermatids (RS), respectively. The spindle assembly checkpoint (SAC) is activated during division (metaphase-to-anaphase transition). Spermiogenesis includes the histone to protamine transition and the differentiation of RS into elongated spermatids (ES) and finally spermatozoa. Adapted from (Vara and Ruiz-Herrera 2022). Numbers between parentheses indicate the diploid (2n) haploid (n) number for each cell type and the number of chromatids per chromosome (4c, 2c, or c). **B** Schematic representation of chromosome pairing and MSCI dynamics during prophase I progression. Description of general patterns is based on evidence in mouse meiosis (adapted from Waters and Ruiz-Herrera 2020). **C** The formation and repair of DSBs occurs in the context of the SC (Adapted from Dapper and Payseur 2019). The proteins central to different steps during the formation and repair of DSBs, including homology search and strand invasion; synapsis; CO and NCO decision; and CO resolution. Briefly, the DSBs machinery is formed by the MCD recombinosome (MEI4-Containing DSB-promoting), a proteinaceous complex that includes MEI4, REC114 and IHO1 (Robert et al. 2016). Once the double-stranded DNA is resected, SPO11 is erased and posteriorly depleted from the recombination site, leaving 3’ single-stranded overhangs at both sites of DSBs (Lam and Keeney 2015). These 3’ single-stranded overhangs are coated by RPA (Robert et al. 2016), which recruits the recombinases RAD51 and DMC1 to facilitate the homologous search of single-stranded DNA filaments. After resected strands have undergone homology searching and strand invasion, DSBs will be repaired either as crossovers (COs) or non-crossovers (NCOs), with each process modulated by different proteins. COs are Holliday junctions (HdJ) resolved by the MutL complex (composed of MLH1 and MLH3), which is recruited by TEX11 (Dapper and Payseur 2019). HEI10 is also recruited to the SC together with the MutL complex, antagonistically regulating RNF212 (Reynolds et al. 2013).

Recombination is essential for sexual reproduction due to its dual role in: (i) assembling new combinations of allelic variants that generate and maintain genotypic diversity; and (ii) establishing physical associations between homologous chromosomes to enable their faithful segregation during meiosis. From extensive work done in model organisms (i.e., yeast, fruit flies, nematodes, mice) and humans it is known that recombination occurs during the first meiotic prophase, which is organised into five stages: leptonema, zygonema, pachynema, diplonema and diakinesis (Fig. 1A). At leptonema, programmed double-strand breaks (DSBs) are generated and homologous chromosomes start to condense, pair and synapse. Chromosomes adhere to the nuclear envelope by their telomeres in the bouquet structure (reviewed in Reig-Viader et al. 2016), prompting the formation of the proteinaceous structure of the synaptonemal complex (SC) that, together with meiotic cohesins, acts as a scaffold for chromosome synapsis and recombination.

The formation of DSBs activates the DNA damage response (DDR) mechanism (Baudat et al. 2010; Myers et al. 2010; Parvanov et al. 2010), an integral part of the meiosis programme. Both DSB formation and DDR are tightly regulated by meiotic checkpoints, including (i) the response to unrepaired DSBs; (ii) transcriptional repression called meiotic silencing of unsynapsed chromatin (MSUC); and (iii) the spindle assembly checkpoint (Subramanian and Hochwagen 2014) (Fig. 1A). Sex chromosomes are subjected to transcriptional silencing during the first male meiotic division by a phenomenon called meiotic sex chromosome inactivation (MSCI), a sex chromosome-specific extension of MSUC (Turner 2005) (Fig. 1B). This is observed in pachynema spermatocytes as the ‘sex body’, which is enriched for repressive histone modifications (Handel 2004).

Importantly, the SC establishes the chromosomal context in which synapsis and recombination between homologues take place (Fig. 1C). The successful progression of early prophase I is dependent on the assembly of chromatin loops into chromosomal axes and the formation and repair of DSBs (Keeney et al. 1997; Romanienko and Camerini-Otero 2000; Longhese et al. 2009). Crucially, the higher-order meiotic chromosome structure regulates the number and distribution of DSBs, and hence the final number of crossovers (COs) per cell (Zickler and Kleckner 1999; Kleckner 2006; Vara et al. 2021). This, in turn, results in a close interplay between SC length and DNA loop size, influencing CO distribution (Zickler and Kleckner 1999; Kleckner 2006). So it appears that SC axis length is a quantitative characteristic of synapsis that is strongly associated with recombination rate (Ruiz-Herrera et al. 2017; Wang et al. 2019). Importantly, in humans, the varied recombination rates within and between individuals have been linked to differences in SC length (Lynn et al. 2002). This was later confirmed in other taxa (Ruiz-Herrera et al. 2017; Wang et al. 2019), with a potential impact on the evolution of recombination rates (Wang et al. 2019; Sardell and Kirkpatrick 2020).

## Patterns of variation in recombination rates

As meiotic recombination influences genome evolution, mammalian recombination landscapes are a reflection of the selective forces that affect the DNA sequence itself, the chromosomal distribution of COs (see below), and the three-dimensional genome folding in germ cells (Vara et al. 2021; Vara and Ruiz-Herrera 2022). Traditionally, theoretical work on the evolution of recombination rates has outnumbered the empirical evidence of recombination variation, especially in natural populations. This was mainly due to the intrinsic difficulties of directly measuring recombination events. However, this has improved over recent years as different approaches have been developed to estimate the number and genomic distribution of COs. These approaches can be classified as direct measurements (i.e., direct measure of recombination events or DSB sites in meiotic cells; Pan et al. 2011; Dumont and Payseur 2011; Smagulova et al. 2011; Brick et al. 2012; Segura et al. 2013; Fowler et al. 2014; Ruiz-Herrera et al. 2017) or indirect measurements of recombination (i.e., estimation of recombination rates using linkage data; Ellegren et al. 2012; Chan et al. 2012; Munch et al. 2014; Sparks et al. 2019; Turbek et al. 2021).

Empirical studies using both direct and indirect approaches have described variation in recombination rates within meiotic cells of the same individual, between individuals, populations, sexes and species, influencing patterns of heritability (Capilla et al. 2016; Stapley et al. 2017; Wang et al. 2019; Vara et al. 2021; Johnston et al. 2016; Kawakami et al. 2019). DSBs induced in early prophase I are higher in number than the final CO number, in some cases substantially more (≥10-fold) (Cole et al. 2012). The DSBs:COs ratio can vary between species, from 10:1 in mice to 3:1 in carnivores (Segura et al. 2013), likely influencing the observed differences in recombination rates.

**Fig. 2:**
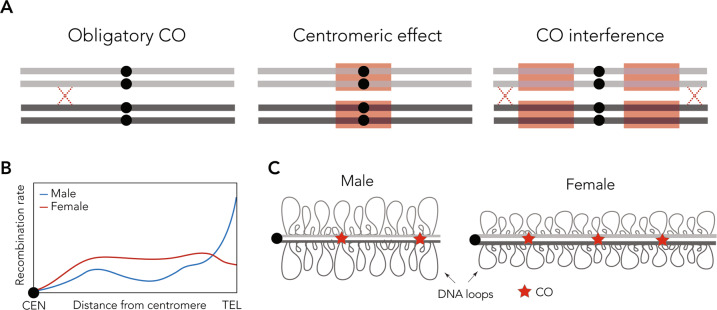
Crossover patterning. **A** Specific features that reduce local frequency (red box) of COs (red crosses). Chromosomal distribution of COs is influenced by the obligatory CO, centromeric effect and CO interference. **B** Schematic representation of general patterns of heterochiasmy in eutherian mammals with high recombination rates in females. **C** Chromosomes with short chromosomal axes (males) tend to show longer DNA loops than those with long chromosomal axes (females). These longer axes and shorter DNA loops in females result in increased COs (red stars). CO crossover, CEN centromere, TEL telomere, CO crossovers.

Early genetic studies (Carpenter 1988) noticed that COs were non-randomly distributed along chromosomes (i.e., recombination hotspots), which were later identified by the immunodetection of MLH1 (Baker et al. 1996; Anderson et al. 1999; Lynn et al. 2002). After decades of study in different organisms it is currently accepted that the chromosomal distribution of COs exhibits four specific features, which are conserved in most species (Fig. 2A): (i) the presence of an obligatory CO; (ii) the phenomenon of CO interference; (iii) the centromeric effect; and (iv) CO homeostasis.

(i) Obligatory CO: there is normally a minimum of one CO per chromosome arm or chromosome, the so-called obligatory chiasma (Bishop and Zickler 2004; Zickler and Kleckner 2015). This serves to establish a necessary physical connection between homologous chromosomes during prophase I to avoid aneuploidies after chromosome segregation. This is a widely conserved pattern in eukaryotes (Zickler and Kleckner 1999; de Villena and Sapienza 2001; Segura et al. 2013).

(ii) CO interference: when a CO forms at one site of the chromosome this interferes with the establishment of COs at adjacent sites due to ‘interference’, resulting in evenly spaced COs (Muller 1916; Kleckner et al. 2003; Wang et al. 2015). This process is pervasive, and although the mechanisms behind it are not currently fully understood, two types of models to explain CO interference: mechanical models and diffusion-based models (reviewed in von Diezmann and Rog 2021).

(iii) Centromeric effect: centromeres normally act as ‘cold’ regions, with a reduction of COs (Beadle 1932; Mather 1939; Cappelletti et al. 2019). Low rates of recombination at centromeres were initially described in *Drosophila* using genetic maps, and later confirmed in different taxa ranging from plants to humans (Lynn et al. 2002; Colome-Tatche et al. 2012). The mechanisms governing centromere effect are far from understood, but this conserved phenomenon probably indicates the presence of strong selective constraints to avoid disruption of pericentric sister chromatid cohesion (Talbert and Henikoff 2010).

(iv) CO homeostasis: this phenomenon buffers the system against deficits (and excesses) of DSBs in meiocytes (Martini et al. 2006; Yokoo et al. 2012; Cole et al. 2012; Wang et al. 2015). It was initially defined as the maintenance of CO frequency even though precursors of DSBs are fewer (Martini et al. 2006). CO homeostasis balances the ratio between CO and NCOs to maintain the obligatory CO on each chromosome. This homeostasis is mechanistically linked to CO interference. As such, COs are maintained in a given cell at the expense of NCOs (Martini et al. 2006).

In addition to the four features described above, sex can also influence recombination rates. Heterochiasmy (sexual dimorphism in recombination rates) has been reported in many taxa, from invertebrates to mammals (Morgan 1912; Lynn et al. 2002). Early cytological work on human meiocytes observed a co-variation between SC length and recombination rates in both sexes (Wallace and Hultén 1985; Baker et al. 1996; Lynn et al. 2002) (Fig. 2B). SC length is longer and CO numbers are higher in oocytes than in spermatocytes (Wang et al. 2017), resulting in higher recombination rates in females (Fig. 2C). Increased SC length and heterochiasmy has also been observed in mouse (Lynn et al. 2002), zebrafish (Wallace and Wallace 2003), planarian (Jones and Croft 1989) and plants (Drouaud et al. 2007; Capilla-Pérez et al. 2021).

We have described that it is important to consider both the cellular context and the molecular constraints underlying the genomic distribution and frequency of meiotic recombination. But, in order to understand the interplay between sex, recombination and the meiotic programme, we need to explore Y chromosome evolution.

## Theories of Y chromosome evolution

Although the current therian X and Y chromosomes have very different structure and gene content, they were once an ordinary pair of autosomes (Ohno 1967). In the therian ancestor (approximately 180 MYA), the proto-Y obtained the testis determining gene *SRY* (Foster et al. 1992). It has been hypothesised that once this new sex-determining system was established, male beneficial alleles accumulated nearby (in linkage disequilibrium) so that they were more likely inherited in males. Ultimately recombination was suppressed between the X and Y across this region, generating the first male-specific region of the Y (Rice 1996). This absence of recombination signalled the initial demise of Y-borne gene function, leading to its degradation.

The mechanisms leading to suppressed recombination have been extensively debated in the literature (Kratochvíl et al. 2021). It was long thought that inversions on the Y resulted in large regions of recombination suppression in single events (Lahn and Page 1999). However, other mechanisms for suppression of recombination have been proposed, including (i) pre-existing low recombination rates on autosomes that become sex chromosomes (Bergero et al. 2019; Rifkin et al. 2021; Xue et al. 2021); (ii) gradual expansion of suppressed recombination rather than large stepwise suppression (Darolti et al. 2020); (iii) different reproductive strategies (Mackiewicz et al. 2018); and (iv) even a neutral model of suppressed recombination (Jeffries et al. 2021). These models are not necessarily mutually exclusive, with different sex chromosome systems likely losing recombination via one or more of these mechanisms (Kratochvíl et al. 2021). Irrespective of how recombination was suppressed, in mammals Y degradation followed, resulting in the sex-specific chromosome becoming a relic of its former self.

Once thought to be a dominant element in the genome (because of its dominant testis determining action), the current degenerated nature of mammalian Y suggests that it is largely a wimpy relic of the X (the so-called wimpy Y—reviewed in Marshall Graves (2000)) (Table 1). Y chromosome decay has not been linear; instead, there were waves of gene loss, presumably from new male-specific regions of the Y soon after recombination was suppressed with the X (reviewed in Charlesworth 2021).

**Table 1:** Models of Y evolution.

Hypothesis	Main features	Prediction	Reference
Dominant Y	Dominant testis determining chromosome	Y chromosome remains as carrier of sex-determining genes	Reviewed in Marshall Graves (2000)
Selfish Y	Accumulates genes beneficial to males	Y chromosome remains as attractor for selfish growth factors	Reviewed in Marshall Graves (2000)
Wimpy Y	Degraded relic of the X having lost almost all genes	In the advent that Y chromosome no longer bears any genes required for general functions it will be lost	Aitken and Marshall Graves (2002)
Fragile Y	Small PARs result in aneuploid gametes, resulting in pressure to remove gene function from the Y	Taxa that evolve achiasmatic segregation during male meiosis will rarely lose the Y chromosome	Blackmon and Demuth (2014, 2015)
Persistent Y	Meiotic executioner genes are required for MSCI, and then must be silenced by this mechanism	Only rare transposition events to the X, where they remain subject to obligate meiotic silencing, are heritable, posing strong evolutionary constraint for the Y chromosome to persist	Waters and Ruiz-Herrera (2020)

**Fig. 3:**
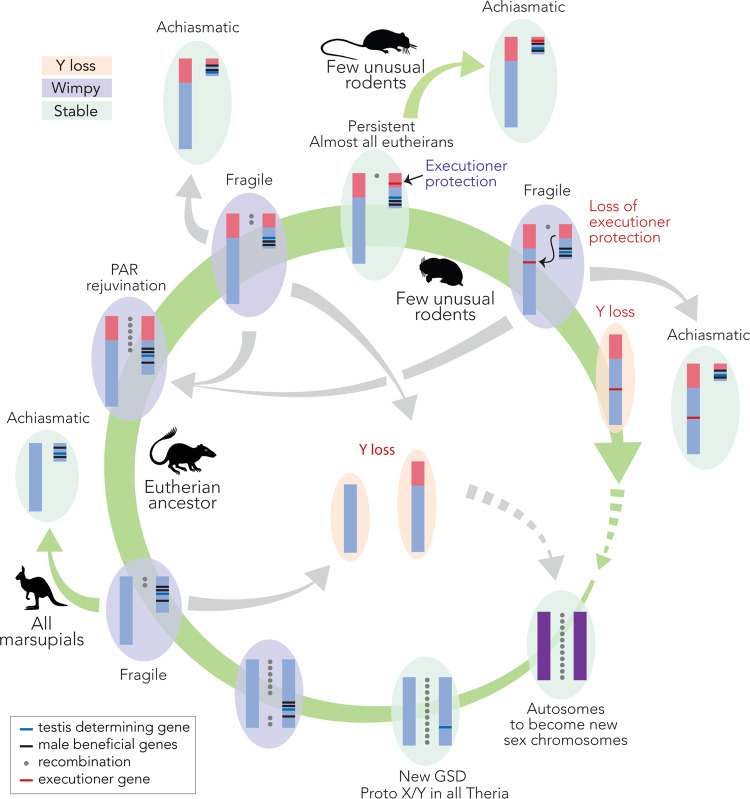
Circular nature of therian Y chromosome evolution. Initially, an ordinary pair of autosomes (purple chromosomes) became the therian sex chromosomes (blue chromosome). The proto-Y acquired a testis determining allele (new genetic sex determination [GSD], blue horizontal line) in the therian ancestor, around which male beneficial alleles accumulated (black horizontal lines). Recombination (grey circles between chromosome pairs) was suppressed across this region, signalling that the Y had become wimpy (purple ovals). As PAR size is reduced, the Y becomes fragile. Once a Y is fragile, there are four possible trajectories of evolution: (i) achiasmatic XY meiotic pairing (i.e., all marsupials); (ii) PAR rejuvenation (i.e., addition of pink autosomal material to the PAR in the ancestor to all eutherians); (iii) Y loss; or, (iv) gaining executioner protection (red horizontal line) to become persistent (i.e., still observed in almost all eutherians). Executioner protection could evolve on the Y at any stage that it is fragile; however, it is only shown after rejuvenation of the PAR in the eutherian ancestor. Persistent Y chromosomes, and those with achiasmatic meiotic pairing, are stable with no observation of being lost (green ovals). In a few unusual rodents (e.g., *Lasiopodomys mandarinus*, *Dicrostonyx torquatus*, *Myopus schistocolor*, *Mus minutoides*; Gil-Fernández et al. 2021; Saunders and Veyrunes 2021), persistent Ys have gained achiasmatic meiotic pairing. In a few other unusual rodent groups (e.g., *Ellobius* and Ryukyu spiny rats; Arakawa et al. 2002; Matveevsky et al. 2017) executioner protection has been removed, resulting once more in a wimpy and fragile Y, permitting its loss in some species (orange ovals). In these species, different autosomes must have become new sex chromosomes (either XY or ZW), completing the cycle. Green arrows represent evolutionary trajectories that have been observed in different mammal groups. Where there are two green arrows exiting a node, the groups of mammals that those trajectories have occurred in are shown. Grey arrows are proposed evolutionary trajectories despite a lack of observation in nature. Dashed arrows represent the completion of the circle, with a new pair of autosomes to become sex chromosomes.

Y chromosomes have also been proposed to be fragile (Table 1) (Blackmon and Demuth 2014, 2015). Under the fragile Y hypothesis, a small PAR results in a less faithful pairing of the X and Y during male meiosis, subsequently increasing the stress of aneuploid gamete production as a result of improper segregation. This may impose a selective pressure to remove functions (i.e., genes) from the Y chromosome, predisposing it to being lost from the population. To prevent this aneuploidy stress and movement of function away from the Y, faithful achiasmatic mechanisms for XY segregation needs to evolve. In marsupial, a dense plate structure, rich in SC proteins, ensures faithful segregation in the absence of synapsis and recombination during the first meiotic division (Page et al. 2003; Marín-Gual et al. 2022). Alternatively, the PAR can be rejuvenated (extended) by the addition of autosomal material, as occurred in the eutherian ancestor under the addition-attrition hypothesis (Graves 1995). However, as new regions of the PAR stop recombining, PAR size is reduced and the Y degrades further, becoming fragile once more. PAR rejuvenation is only a temporary reprieve from fragility. So, in the absence of achiasmatic sex chromosome segregation, it appears that loss from the population is the inevitable fate of Y (or W) chromosomes in heteromorphic sex chromosome systems (Fig. 3).

However, Y chromosomes remain in all but a few therian mammal species (i.e., Ryukyu spiny rat and some *Ellobius* species) (Arakawa et al. 2002; Matveevsky et al. 2017; Furman et al. 2020; Vicoso 2019) since it arose approximately 181 MYA (Cortez et al. 2014). Indeed, the current human Y is relatively stable (Bellott et al. 2014, 2017; Cortez et al. 2014). This contrasts with observations in other vertebrate clades, in which there is regular sex chromosome turnover (Ezaz et al. 2006, 2010). In amphibians, there are some frogs with at least 13 sex chromosome turnover events in 28 species, which occurred within approximately 55 million years (Jeffries et al. 2018).

## Persistent Ys in the meiotic context

Meiosis in general, and recombination (or the absence of it) more specifically, are both key elements to sex chromosome evolution. However, a unifying framework that accounts for how the meiotic cellular programme can influence sex chromosome evolution is still missing. This has been mainly due to the intrinsic difficulties of directly measuring recombination events and monitoring meiosis progression in germ cells of key representative phylogroups. So, despite being perceived as a fragile wimpy, why does the Y chromosome persist across large evolutionary time scales in the majority of mammalian species? Here we present an integrative view on how the meiotic programme and its mechanistic constraints can shape the evolution of differentiated sex chromosomes. We propose that the persistence of Y chromosomes in different phylogroups can be explained in the context of several interrelated aspects of sex chromosome biology and meiosis: (i) variation in PAR size; (ii) high recombination rates in the PAR to ensure proper segregation; and (iii) MSCI and the presence of Y-borne executioner genes.

### Variation in PAR size: implications for Y evolution

In eutherian mammals, the PAR represents a small portion of the Y chromosome. PAR rejuvenation occurred in the eutherian ancestor (after marsupial divergence, but pre-eutherian radiation) under the addition-attrition model of sex chromosome evolution (Graves 1995). After PAR extension by this autosomal addition (Fig. 3), recombination was further suppressed between the X and Y. Different rates of Y degradation and recombination suppression within phylogroups resulted in variation of PAR size among mammals, from ≈700 Kbp in mice to ≈10 Mbp in alpaca (Raudsepp et al. 2012). In some eutherian clades (e.g., some bat, rodents, bovids and primates) the PAR has been rejuvenated, (Murata et al. 2012; Britton-Davidian et al. 2012; Rahn et al. 2016; Vozdova et al. 2016) presumably resulting in new Y specific material after the suppression of recombination between the X and Y in the extended PAR.

Variation in PAR size is an important driver for Y evolution under the fragile Y hypothesis (Blackmon and Demuth 2014, 2015) (Table 1). Y losses in the offspring (i.e., XO individuals) are frequently found in species with small PARs (0.7–2.7 Mb, i.e., human, mouse and horse; reviewed in Raudsepp and Chowdhary 2016) with negative (often fatal) consequences for the individual if not mosaic as reported in human and horse (Raudsepp et al. 2012). This aneuploidy stress would put pressure on the critical functions to be moved from the Y to the autosomes, as has been observed in mammals (Hughes et al. 2015).

In contrast, in domestic species with larger PARs (>5 Mb, i.e., ruminants, pigs and carnivores; reviewed in Raudsepp and Chowdhary 2016) XO individuals are infrequently observed, potentially as a result of more haploinsufficient genes being lethal (Raudsepp et al. 2012). However, it is tempting to speculate that under the fragile Y hypothesis, a larger PAR results in more faithful pairing and segregation of the sex chromosomes, so the generation of Y-less gametes is less frequent. Or perhaps a combination of both results in the observation of fewer XO individuals. That is, larger PARs result in less mis-segregation and a more lethal haploinsufficiency effect.

### Recombination in the PAR

Progressive degeneration of the Y chromosome has led to the evolution of meiotic mechanisms that ensure faithful XY segregation during male meiosis. In eutherian mammals, most species have a PAR (reviewed in Raudsepp and Chowdhary 2016) that has evolved at high rates of recombination to ensure an obligate CO during male meiosis (Kauppi et al. 2011). In contrast to autosomes where DSBs occur every 10 Mbp (Kauppi et al. 2011), PARs are characterised by a recombination rate 10–20-fold higher than the genome average (e.g., 1–2 DSBs every 1 Mbp in rodents) (Kauppi et al. 2011). Initial studies proposed that this prevalence of DSB formation in the PAR was induced by a high-order chromosome structure distinct from that of the autosomes, such as the presence of abundant short DNA loops in a relatively long chromosomal axis (Kauppi et al. 2011). This was later demonstrated by the accumulation of *cis*- and *trans*-acting DSB-promoting proteins in the PAR during early prophase I (Papanikos et al. 2019; Acquaviva et al. 2020). Whether this observation in mouse holds for other eutherian mammals remains to be experimentally demonstrated. However, evidence of a correlation between DSBs, DNA loop size and chromosomal axes length in different taxa (Ruiz-Herrera et al. 2017; Wang et al. 2019) suggests this to be the case.

We propose that in species where the heterogametic sex is achiasmate (that is, sex chromosomes do not pair and recombine), the absence of a PAR would not impose selective pressure to initiate excessive numbers of DSBs genome wide to ensure a CO in a small PAR. Marsupials, which lack a PAR, are characterised by some of the lowest recombination rates within mammals (Zenger et al. 2002; Segura et al. 2013) and low number diploid numbers (which range from 2n = 10 to 2n = 32, Deakin and O’Neill 2020). These low levels of recombination in marsupials could result from a reduction of DSB formation genome wide (Marín-Gual et al. 2022). As a result of no PAR, DSBs are only required at a frequency sufficient to result in obligatory COs on the few large autosomes.

### MSCI and the presence of Y-borne executioner genes

Whether the heterogametic sex is achiasmate (i.e., marsupials and some rodent species), or not (i.e., eutherian mammals), MSCI occurs and is a necessary checkpoint to ensure proper meiotic progression. It was recently proposed that the unique features of MSCI might explain the persistence of the Y chromosome in most eutherian species (Waters and Ruiz-Herrera 2020). That is, Y-borne meiotic executioner genes, integral to meiotic checkpoints, lead to the persistence of Y chromosomes in species with MSCI (i.e., a persistent Y, Table 1). Meiotic executioners are pachytene lethal if they escape MSCI (Royo et al. 2010; Vernet et al. 2016), resulting in the production of inviable gametes. In addition to executing the cell when ectopically expressed, to be a truly selfish element that protects the Y, executioners are also required for the initiation of MSCI (Royo et al. 2010; Vernet et al. 2016). The result is a gene that has evolved to become its own sensor.

Under the persistent Y hypothesis (Waters and Ruiz-Herrera 2020), once a Y chromosome is under execution protection, it cannot be lost from the population. The Y as a unit is required for successful MSCI, to which it must then be subject. Under no circumstance can viable Y-less gametes be produced. Should an executioner be translocated to an autosome, MSCI proceeds but the executioner escapes silencing and the cell dies. The only location in the genome to which an executioner gene can move is the X chromosome. From there it must retain its function to properly initiate MSCI, so that it is appropriately silenced, permitting meiosis to proceed. Indeed, the movement of executioner genes from the Y to the X preceded Y chromosome loss in Y-less rodent species (reviewed in Waters and Ruiz-Herrera 2020). In eutherian mammals, *Zfy* acts as the executioner, and its translocation to an autosome explains complete azoospermia reported in a horse (Ruiz et al. 2019; Bugno-Poniewierska and Raudsepp 2021). It is important to note that persistent Ys are only possible in clades with MSCI already established, providing a haven from which genes can evolve a selfish executioner function.

The evolution of executioner function is a fascinating question, especially in light of the fact that *Zfy* was part of an addition to the sex chromosomes in the eutherian ancestor, and remains autosomal in marsupials (Waters et al. 2001). Being a pachytene lethal executioner gene, it is no surprise that *Zfy* expression is low in the spermatocytes of representative eutherian species (Murat et al. 2021). However, what function could it play from an autosome in non-eutherian species?

In opossum, platypus and chicken, *Zfy* is annotated as its X homologue, *Zfx*. In each of these species, *Zfx* expression is maintained in spermatocytes (Murat et al. 2021), suggesting that the *Zfx*/*y* precursor was not pachytene lethal. Therefore, it could only have gained this aspect of executioner function after arriving on the eutherian Y chromosome. It is unclear if the *Zfx*/*y* precursor already had a function in initiating MSCI before its translocation to the Y; however, it is tempting to speculate that it did. In opossum and platypus, in which MSCI occurs, *Zfx* expression is elevated in spermatocytes compared to other cell types (Murat et al. 2021).

In contrast, *Zfx* expression in chickens is only elevated in pre-meiotic cells (i.e., spermatogonia). But since birds have a ZW sex chromosome system and males are the homogametic sex, there is no requirement for MSCI in the testis. Because MSCI is a prerequisite for the evolution of executioner genes, further studies on the germline of the heterogametic sex in distantly related vertebrate linages will provide important insight into the potential for the evolution of executioner function.

## Closing the circle

Here we have described how sex chromosomes result in exceptions to Mendelian inheritance, highlighting the importance of functional and mechanistic meiotic constraints on sex chromosome evolution. Under a dynamic (and possibly cyclical) evolutionary trajectory (Fig. 3) we propose that Y persistence can be explained in the context of recombination rates, PAR size and Y-borne meiotic executioner genes that regulate MSCI.

Due to suppressed recombination, the PAR represents a small portion of the X and Y. We propose that these fragile Ys with small PARs result in an increase of DSBs (and hence recombination rates) to ensure a CO event in the PAR, and reduce the deleterious effect of aneuploidy. If PAR size is reduced so that the Y becomes too fragile and aneuploidy stress causes loss of Y function, the Y chromosome can become stable by (i) temporary PAR rejuvenation, (ii) evolving achiasmatic XY segregation, or (iii) acquiring executioner protection (Fig. 3). A persistent Y will remain under strong constraints to maintain high recombination rates in the PAR to ensure an obligatory CO, as losing the Y will have fatal consequences for germ cells (i.e., aberrant MSCI). Almost all eutherian Y chromosomes are at this stage. In the rare event that executioner protection is lost (i.e., translocation away from the Y chromosome to the X), the Y chromosome can be maintained in the population by either PAR rejuvenation or evolving achiasmatic XY segregation, the alternative is Y loss.

Under this dynamic cyclic evolutionary scenario, the intricacies of the meiotic programme influence the fate of Y chromosomes. Understanding the progression and regulation of the meiotic programme in vertebrate and invertebrate species will be important to further decipher the plasticity of the rise and fall of heteromorphic sex chromosomes.
